# Pediatric non alcoholic fatty liver disease: old and new concepts on development, progression, metabolic insight and potential treatment targets

**DOI:** 10.1186/1471-2431-13-40

**Published:** 2013-03-25

**Authors:** Valentina Giorgio, Federica Prono, Francesca Graziano, Valerio Nobili

**Affiliations:** 1 Hepato-metabolic Unit, Bambino Gesù Children Hospital, Rome, Italy

**Keywords:** Nonalcoholic fatty liver disease, Non-alcoholic steatohepatatis, Obesity, Metabolic syndrome

## Abstract

Nonalcoholic fatty liver disease (NAFLD) is the leading cause of chronic liver disease in children. NAFLD has emerged to be extremely prevalent, and predicted by obesity and male gender. It is defined by hepatic fat infiltration >5% hepatocytes, in the absence of other causes of liver pathology. It includes a spectrum of disease ranging from intrahepatic fat accumulation (steatosis) to various degrees of necrotic inflammation and fibrosis (non-alcoholic steatohepatatis [NASH]). NAFLD is associated, in children as in adults, with severe metabolic impairments, determining an increased risk of developing the metabolic syndrome. It can evolve to cirrhosis and hepatocellular carcinoma, with the consequent need for liver transplantation. Both genetic and environmental factors seem to be involved in the development and progression of the disease, but its physiopathology is not yet entirely clear. In view of this mounting epidemic phenomenon involving the youth, the study of NAFLD should be a priority for all health care systems. This review provides an overview of current and new clinical-histological concepts of pediatric NAFLD, going through possible implications into patho-physiolocical and therapeutic perspectives.

## Introduction

Nonalcoholic fatty liver disease (NAFLD) has recently emerged as the leading cause of chronic liver disease in children [1-3]. The increasing incidence of NAFLD is a mirror of the worldwide annual increment of obese individuals [4-6]. Data from the last National Health Nutrition Examination Survey shows that 17% of all children in western countries are overweight. Among them, 70% to 80% have NAFLD.

NAFLD is defined by hepatic fat infiltration >5% hepatocytes, as assessed by liver biopsy, in the absence of excessive alcohol intake, or the evidence of viral, autoimmune or drug-induced liver disease. It includes a spectrum of liver disease ranging from intrahepatic fat accumulation (steatosis) to various degrees of necrotic inflammation and fibrosis (non-alcoholic steatohepatatis [NASH]) [7,8]. Pediatric NAFLD histopathology has distinct characteristics when compared with that of adults [9], but whether these differences are due to different mechanisms in the pathogenesis, or represent two phenotypes, is still unclear.

Despite the differences, pediatric NAFLD is associated, as it is in adults, with severe metabolic impairments such as insulin resistance, hypertension and abdominal obesity, determining an increased risk of developing type 2 diabetes mellitus, the metabolic syndrome and cardiovascular diseases [10,11]. The natural history and prognosis of NAFLD in children is still uncertain, since published data with long-term follow up is scant. It has been reported that, in susceptible individuals, NAFLD can evolve to cirrhosis and hepatocellular carcinoma, with the consequent need for liver transplantation [12]. Both genetic and environmental factors seem to be involved in the development and progression of the disease [13,14], and a better understanding of its physiopathology will certainly help in reducing future morbidity and mortality from NAFLD. Moreover, in view of this mounting epidemic phenomenon involving the youth of today, the study and identification of treatment for children and adolescents with this liver disease should be a priority for all health care systems.

This review will therefore provide an overview of the current concepts in epidemiology, histological features, etiopathogenesis, and the diagnosis and treatment of pediatric NAFLD, but will particularly aim to go through the new concepts in the aforesaid field and their possible implications into patho-physiolocical and therapeutic perspectives.

### Epidemiology and risk factors

Data published from centers in North America, Europe, Asia, South America and Australia [15-17] demonstrates that NAFLD nowadays interests not only the western countries. Pediatric NAFLD prevalence is estimated to be between 3% and 10%. It varies as such because it is influenced by the characteristics of the population, especially lifestyle habits, and the diagnostic method used to detect it. In fact, although liver histology is the gold standard for diagnosing NAFLD, performing biopsies to determine disease prevalence is not always feasible. Children with NAFLD typically have slightly elevated liver enzyme values (aspartate aminotransferase [AST], and alanine aminotransferase [ALT]) in the absence of excessive alcohol consumption and other causes of steatosis [18]. Therefore, elevated serum levels of liver enzymes, even though they often misrepresent the entity of intrahepatic damage, are used as a valuable and noninvasive test to screen for pediatric NAFLD [19]. Alternatively, BMI and ultrasonography can also be used, even though their diagnostic accuracy limitations are the same as those reported for ALT (for instance, fibrosis is undetectable with these methods).

One of the few population-based studies published in this field reports data from the United States (NHANES III) [20] showing that the prevalence of elevated ALT levels (> 30 IU/L) was 7.4% among white adolescents, 11.5% among Mexican American adolescents and 6.0% among black adolescents. Elevated ALT levels were present in 12.4% of male subjects compared with 3.5% of female subjects. Data from South Korea [16] and Japan [21] is similar. It is, therefore, reasonable to assume that NAFLD prevalence in adolescents is at least 2.6% to 3.2%. As a matter of fact, this data underestimates the real prevalence, since surrogate markers have been used to diagnose NAFLD.

An autopsy study by Schwimmer and colleagues [22] reviewed the records and liver histological features of 742 children aged 2 to 19 years, who died from unnatural causes in San Diego County between 1993 and 2003. The prevalence of fatty liver (> 5% of hepatocytes containing macrovesicular fat), adjusted for age, gender, race, and ethnicity was estimated to be 9.6%, although the autopsy cohort was understandably biased relative to the general population, due to overrepresentation of Hispanic boys.

Despite the diversity of diagnostic criteria used in population-based studies, obesity is the main risk factor for pediatric NAFLD. In fact, the prevalence of NAFLD in obese children increases up to 80% in several obesogenic countries, including USA, Europe and Japan [21-26]. Huang et al. [27] recently reported that in 219 schoolchildren, aged 6 to 12 years, rates of NAFLD were 3% in the normal-weight range, 25% in the overweight range, and 76% in obese children.

A population-specific based study [28] has been performed in Europe. One-hundred-and-eleven specialized pediatric obesity centers in Germany, Austria and Switzerland were involved, and 16,390 overweight, obese and extremely obese children and adolescents were included and studied for NAFLD. NAFLD was defined by AST and/or ALT > 50 Ul-1. NAFLD was present in 11% of the study population, predominantly in boys (boys vs girls; 14.4:7.4%; P<0.001), and in the extremely obese group (obese vs extremely obese; 9.5:17.0%; P<0.001).

NAFLD in children is also strongly associated with several other features of the metabolic syndrome (MS), especially insulin resistance and type 2 diabetes mellitus [29,30]. It also increases the risk of developing cardiovascular disease in adulthood. A recent cohort of pediatric patients has been studied for carotid intimal medial thickness as a marker of atherosclerosis. This was significantly greater in obese children diagnosed with NAFLD, than that in the control group, indicating that obese children with NAFLD are associated with a greater risk of atherosclerosis and future adverse cardiovascular events [31].

Interestingly, it has been reported that 20–80% of children with NAFLD can be presented and associated with hypertriglyceridemia and/or hypercholesterolemia [32,33]. In particular, the prevalence of NAFLD increases in hyperglycemic patients, and insulin resistance is more severe in individuals with NASH, than in those with simple steatosis. Our group evaluated a cohort of 120 Italian children (3–18 years) with biopsy-proven NAFLD or NASH for correlations between clinical or biochemical variables and liver histology. Dyslipidemia was largely diagnosed - low HDL cholesterol was found in 45% of the population, and hypertriglyceridemia in 63%. The presence of MS or clinical and biochemical variables associated with the syndrome were strictly related to histological features of NASH.

It is well documented that NAFLD can occur in very young children, but it is more prevalent in adolescents [34]. Factors that can explain the higher rate of NAFLD in adolescents include sex hormones and insulin resistance in puberty [35,36], or their increased control over unhealthy food choices and sedentary lifestyle [37].

It is more common in boys than in girls [35] with a male to female ratio of 2:1. It is possible to hypothesize that estrogens can be potentially liver-protective; or indicate that androgens may aggravate NASH [38,39].

Ethnicity can also affect the prevalence of NAFLD and has been consistently investigated across studies involving largely multiethnic populations. NAFLD is more common in Hispanic than in Caucasian children [20,35,40]. Ethnic differences could possibly be due to higher rates of insulin resistance, and to visceral adiposity at equivalent body mass index (BMI), but also as a result of socio-economic factors, including type of diet, exercise choice and living location. Despite the fact that African- American children often present more risk factors for NAFLD—including obesity, insulin resistance and type 2 diabetes mellitus—the rate of NAFLD in these individuals is lower [41]. It is an area of intense research.

### Histopathological pattern and New scoring systems

The pattern of the distribution of pediatric NAFLD histological lesions is frequently different from that of adults. Classical histological findings that characterize NAFLD in children are: steatosis, ballooning, inflammation and fibrosis [7]. These features have been deeply characterized elsewhere [13]. Evidence of steatosis in >5% of hepatocytes is the necessary criterion for diagnosing NAFLD both in adults and children [42]. Steatosis distribution is the first distinctive feature between adult and pediatric NAFLD. In adults, steatosis starts in the perivenular zone (acinar zone 3), whereas in children, it typically starts in the periportal zone (acinar zone 1) or shows azonal distribution. The reason why these 2 different patterns are described as such is still unknown [43,44]. Inflammatory infiltrate (including lymphocytes, histiocytes or Kupffer cells) can be present in lobules or in portal tracts of the liver [7]. Hepatocyte ballooning is a major distinguishing feature of NASH that confers an increased risk of disease progression [45]. The importance of ballooning has been emphasized by the results of the two largest randomized trials for NAFLD treatment in adults and children, namely The TONIC and The PIVENS trial [46,47]. Fibrosis of the liver occurs as a response to the insult of NASH. As for steatosis, in children, the fibrosis pattern is prevalent in zone 1.

In 2005, Schwimmer et al. [48] categorized two prevalent patterns of NASH in children: the ‘adult type’, in which steatosis has a zonal distribution prevalent in zone 3, and is associated with lobular inflammation, ballooning and perisinusoidal fibrosis; and the ‘pediatric type’, in which steatosis is associated with portal inflammation and portal fibrosis in the absence of ballooning. Other studies have more recently found that these patterns can overlap in variable degrees of combination [49]. The National Institute of Diabetes and Digestive and Kidney Diseases and the NASH Clinical Research Network (CRN) Pathology Committee introduced, above the categories of ‘not-NASH’ and ‘definite-NASH’, the categories of ‘borderline zone 1 pattern’, to describe a NASH type with prevalent portal alteration, and of ‘borderline zone 3 pattern’ in cases of prevalent alterations in zone 3. The zone 1 NASH pattern is often seen in children [49]. A hypothesis that may explain the heterogeneity in histology patterns of pediatric NASH is that the ongoing pathology may start in zone 1, and result in the adult pattern later in its natural history. Alternatively, pediatric NASH could be a distinct pathology from adult NASH. Further investigations are needed to carefully characterize the disease in children.

Currently, two main scoring systems exist to evaluate histological activity in NAFLD and NASH, and are used both in children and adults. The Brunt score [50], based on the semi-quantitative assessment of macrovacuolar steatosis, ballooning, lobular and portal inflammation, generates three-tier grades of activity: mild or grade 1; moderate or grade 2; severe or grade 3. The other scoring system is the NASH–CRN system [51], that generates a numeric score for grading the disease, the so-called NAFLD activity score (NAS). NAS results from adding together the individual scores for steatosis (0–3), lobular inflammation (0–3), and ballooning (0–2), range from 0 to 8. A NAS score of 1 and 2 corresponds to ‘not NASH’, whilst a score >5 corresponds to ‘definite-NASH’. Activity scores 3 and 4 are the borderline cases. Both scoring systems generate a numeric score for staging fibrosis: stage 1 (perisinusoidal fibrosis); stage 2 (portal–periportal fibrosis); stage 3 (bridging fibrosis); and stage 4 (cirrhosis).

Since the development of the original NAS score, it has soon become evident that classifying pediatric NASH with its distinct histopathological pattern, mainly characterized by the presence of portal-based disease, including portal inflammation (PI), was particularly difficult. As a matter of fact, using a NAS score, only about half of all children could be categorized into a clear cut pattern, while the other half fell into the “borderline” category, supporting the need for a more reproducible scoring system to interpret liver histology in pediatric cases of NAFLD/NASH.

Therefore, our group studied 203 consecutive children [52], with biopsy–proven NAFLD, aiming to develop a new grading of a ‘specific score’ for Pediatric NAFLD, to be used in clinical trials, which took into account the presence of PI. This score was called the Pediatric NAFLD Histological Score (PNHS). Histological features were scored: steatosis (0–3), lobular inflammation (0–3), ballooning (0–2), and PI (0–2). Our results demonstrated that there was an excellent correlation between PNHS scores and the presence of NASH, suggesting that this ‘specific score’ might be considered to be used for the histological grading of pediatric NAFLD.

### Pathogenesis: present and future perspectives

**Figure 1:**
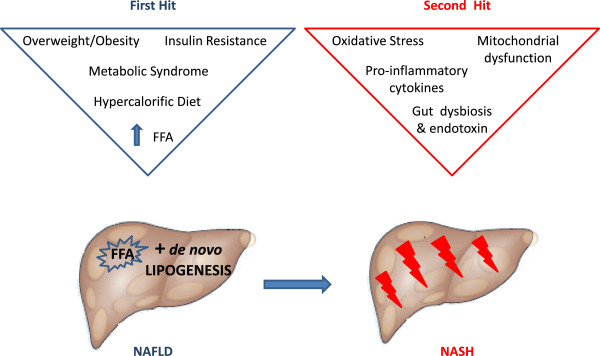
**The “two hits” hypothesis.** Transformation from NAFLD to NASH can be explained by the so called “two hit” hypothesis. The “first hit consists of the intrahepatic accumulation of fatty acids, which is closely associated with insulin resistance, and which increases the susceptibility of hepatocytes to secondary injuries or insults (oxidative stress, mitochondrial dysfunction, overproduction and the release of pro-inflammatory cytokines, and the endotoxin-mediated activation of the innate immune response).

NAFLD occurs in overweight and obese children, in whom the energetic balance, normally based on the perfect equality of income, waste and storage has failed. Multiple factors are probably involved in the pathogenetic mechanisms. All together they create a network of interactions participating in both the development and progression of the disease [13,14], that has been summarized over a decade ago by Day et al. in the so called “two hits hypothesis” (Figure 1). The “first hit” is represented by peripheral insulin resistance, leading to fat accumulation in hepatocytes, and an increased lipid peroxidation. Hyperinsulinemia and insulin resistance, accompanying obesity, lead to liver steatosis, since they increase the absolute free-fatty-acids (FFA) uptake in the liver, the esterification of hepatic FFAs to form triglycerides (TG), the FFA synthesis from cytosolic substrates, and the decreased apolipoprotein B-100 synthesis. Subsequently, the export of FFA and TG is decreased, while the beta-oxidation of mitochondrial long-chain fatty acids is increased. The “second hit” is represented by oxidative stress, which seems to explain the progression to liver fibrosis. Reactive oxygen species (ROS) can induce hepatocellular injury by the inhibition of the mitochondrial respiratory chain enzymes, the inactivation of glyceraldehyde-3-phosphate dehydrogenase and the inactivation of membrane sodium channels. ROS further cause lipid peroxidation, cytokine production, and induce Fas Ligand, contributing to hepatocellular injury and fibrosis. To summarize, the first step consists of the intrahepatic accumulation of fatty acids, which is closely associated with insulin resistance, and which increases the susceptibility of hepatocytes to secondary injuries or insults (oxidative stress, mitochondrial dysfunction, overproduction and the release of pro-inflammatory cytokines, and the endotoxin-mediated activation of the innate immune response). Increased susceptibility to these factors might also explain the progression of NAFLD to NASH [53,54].

Visceral adipose tissue is believed to play a pivotal role in the pathogenesis of NAFLD, since it participates in producing most adipokines, such as Tumor Necrosis Factor alpha (TNF-alpha), resistin, and adiponectin, which are involved in inducing insulin resistance and low-grade inflammation. As a matter of fact, several current therapeutic approaches aim at reducing visceral adipocyte-derived adipokine and FFA overflow to the liver, by weight loss and insulin-sensitizers.

Leptin is a satiety adipokine produced predominantly in adipocytes [55] that regulates appetite and metabolism at the level of the hypothalamus [56]. Most obese humans have high circulating levels of leptin, as a result of what has been characterized as leptin resistance. This phenomenon of leptin resistance may already be present in obese children [57-59]. Adiponectin is an adipose-specific hormone that has anti-inflammatory and insulin-sensitizing properties [60,61]. Similar to adult data, observational pediatric studies have shown an inverse correlation between plasma adiponectin concentrations and adiposity, insulin resistance [62] and hepatic fat [63]. It is promising that adiponectin is favorably modifiable by simple lifestyle changes, and using the levels of total adiponectin, or high-molecular-weight, adiponectin as a biomarker for insulin sensitivity is gaining support. Resistin is another adipokine that antagonizes insulin action, causing glucose intolerance [64] – resistin-deficient animals are protected from obesity – whereas elevated resistin is associated with insulin resistance [65]. Both TNF-alpha and IL-6 are positively related to adiposity, and correlate with insulin resistance and cardiovascular disease (CVD) risk factors [66,67]. They also indirectly mediate lipolysis and augment hepatic synthesis of fatty acids. Studies on the effect of therapies on inflammatory factors are still limited, except for a few studies on IL-6 and TNF-alpha [68-70]. Further interventional trials are awaited to clarify the link among adipokines, NAFLD, MS and CVD.

#### Genetic predisposition

Evidence that only a minority of patients with NAFLD progress to NASH suggests that disease progression is likely to depend on complex interplay between environmental factors and genetic predisposition. The pathogenesis of NAFLD appears to be multifactorial, involving both genetic and environmental factors. Two recent cohort studies and one community-based study in different ethnicities estimated that the heritability of NAFLD could approach 35–40% of the total predisposition, even after adjusting for age, gender, race and BMI [71,72].

Several genetic variants in the genes involved in energy balance, such as adiponutrin/patatin-like phospholipase domain-containing 3 (PNPLA3), and apolipoprotein C3 (APOC3), or in genes involved in inflammation, oxidative stress and fibrogenesis, such as SOD2, have been demonstrated to be associated with NAFLD and the severity of liver damage. The association of the PNPLA3 variant with NAFLD in obese children and adolescents has been deeply characterized. Similarly to obese adults, the PNPLA3 variant confers genetic susceptibility to liver damage as identified by increased ALT and AST liver enzyme levels in young patients. There was no relationship of the PNPLA3 variant with insulin sensitivity, BMI or serum triglycerides, but a close relationship was demonstrated between the PNPLA3 variant and the increased central obesity in obese children, as determined by waist circumference, which contributes to liver damage in children with NAFLD [73].

Since accumulating evidence had suggested the involvement of the endocannabinoid-system in liver disease and related complications, our group [74] has recently studied the hepatoprotective properties for Cannabinoid Receptor type 2 (CB2) in a cohort of 118 obese Italian children with biopsy proven NAFLD. We have found that whereas the CB2 Q63R variant was not associated with steatosis or fibrosis, it was associated with the severity of the inflammation (P = 0.002), and the presence of NASH (P = 0.02), suggesting a critical role for the CB2 Q63R variant in modulating the hepatic inflammation state in obese children, and in the consequent increased predisposition of these patients to develop liver damage.

Several ongoing studies on the genetic predisposition for NAFLD, by different research groups, will certainly and soon help in the identification of new molecular targets for the pharmacological treatment of NAFLD.

### Diagnosis

A recent position paper by the ESPGHAN hepatology committee [75] has clarified the diagnostic approach to NAFLD in childhood, describing the role of different tests, including liver biopsy. NAFLD is more frequent in children aged more than 10 years, and is usually present with overweight/obesity. The diagnosis of NAFLD needs the recognition of fatty liver, and the exclusion of other causes of steatosis. Liver biopsy is the current gold standard for the diagnosis of NAFLD, and it is the only way to distinguish between NASH and simple steatosis, and to determine the severity of liver damage and the presence and extent of fibrosis, as well as to rule out other diagnoses such as autoimmune hepatitis, Wilson disease and metabolic diseases [76]. However, since liver biopsy is an invasive procedure, it is usually not performed as a first step. Primary noninvasive evaluation (biochemical parameters, imaging tests and serum biomarkers) should be used as initial tools to confirm the diagnosis of fatty liver disease, especially in the typical patient with characteristics of MS. The current diagnostic laboratory workup used for the diagnosis of pediatric NAFLD is discussed in Table 1.

**Table 1:** Laboratory work up in suspected pediatric NAFLD

**Laboratory workup**	**What to rule out**
Basic profile: Full blood count, Liver function tests, fasting glucose and insulin, urea and electrolytes, coagulation, INR, iron, ferritin, uric acid	
Lipid profile	Dyslipidemia/Familial hypercholesterolemia/ Cholesterol ester storage disease
Lipoproteins	Abetalipoproteinaemia
Glucose tolerance test (OGTT), glycosylated hemoglobin	Insulin resistance/Type 2 Diabetes Mellitus (DM2)
Thyroid function tests	Hypothyroidism
Ceruloplasmin level	Wilson Disease
Viral hepatitis panel	Viral - hepatitis (HBV, HCV)
C-Reactive-Protein + consider EBV, CMV immune state profile	Acute systemic disease
Sweat test	Cystic Fybrosis
Anti-Transglutaminase IgA and total IgA	Coeliac disease
CPK	Muscular Dystrophy
Alpha-1-antitrypsin serum level	Alpha-1-antitrypsin deficiency
Serum lactate +/− amino and organic acids +/− plasma-free fatty acids +/− acyl carnitine profile	Metabolic diseases (Galactosaemia -in infants-, hereditary fructose intolerance, glycogen storage disease (Type VI and IX), others
Serum Immuniglobulin, Liver autoantibodies	Autoimmune hepatitis
Specific tests as suggested by history, consider:	Drug toxicity, Parenteral Nutrition, Protein malnutrition, others

Laboratory tests in suspected pediatric NAFLD, and correspondent ruled out diseases of other etiology.

Liver function blood tests and imaging techniques (US, CT, MRI) are commonly used as indirect markers of liver steatosis. None of these have proven to be reliable, and the sensitivity and the specificity are undetermined. Aminotransferases are used, together with the measurement of accessible serum parameters such as glucose, triglyceride, cholesterol, lipoproteins, glucose/insulin levels after tolerance tests, and glycated hemoglobin HbA1c (Table 1), to assess NAFLD diagnosis and to screen children from possible hepatopathy-related metabolic complications, such as MS. These measurements must be combined with the evaluation of anthropometric parameters – BMI, abdominal circumference – and with other information such as the patient’s gender and the health state and lifestyle of the relatives. Generally, the AST: ALT ratio is less than 1, but this value can increase as fibrosis progresses. Circulating levels of aminotransferases can fluctuate over time and are normal (<0.67 μkat/l) in a large number of children with NAFLD and NASH [77,78]. Normal levels of serum aminotransferases do not exclude the presence of fibrosis or even cirrhosis. Serum levels of alkaline phosphatase and GGT can also be slightly elevated in pediatric NAFLD. Moreover, positive antinuclear antibody and smooth muscle antibody (SMA) titers can be found in 15.4%, and in 10% of patients, respectively. Positive autoantibodies have been associated with the higher fibrosis stages [79].

Since patients with NAFLD are often present with some components of MS, lipid profiles, as well as fasting glucose and insulin levels, should always be verified [80]. Albumin, bilirubin, and platelet serum levels are usually normal unless the patient is developing cirrhosis.

Identifying and validating potential novel noninvasive biomarkers of NAFLD and NASH is a central area of research. The pediatric NAFLD fibrosis index (PNFI), which is obtained from three simple measurements—age, waist circumference and triglyceride levels—has been developed to predict liver fibrosis in children with NAFLD [81]. This index is easy to calculate with no additional cost to the patient, and it has a good positive predictive value to predict fibrosis; however, its negative predictive value to rule out fibrosis is suboptimal. These limitations can be overcome when used in a sequential algorithm with the enhanced liver fibrosis (ELF) test, which uses a combination of three extracellular matrix components (namely hyaluronic acid, amino-terminal propeptide of type III collagen and TIMP1), resulting in an accurate assessment of the presence of liver fibrosis in children [82]. Future studies are still needed to externally cross-validate these findings, before the combination of PNFI and ELF can be recommended in children with NAFLD. Some evidence has demonstrated the utility of a measurement of plasma levels of a specific byproduct of apoptosis in liver cells—caspase-generated cytokeratin 18 fragments—in the diagnosis of NASH in adults [83]. In 2010, Fitzpatrick *et al.*[84] demonstrated that children with biopsy-proven NAFLD also showed considerably elevated levels of these cytokeratin 18 fragments when compared with healthy controls. These results suggest that measuring cytokeratin 18 fragments could be useful in the work-up of children suspected of having NASH. Larger validation studies are, however, still needed.

#### Imaging techniques

Liver ultrasonography is the most commonly used imaging diagnostic modality, largely because it is relatively inexpensive, widely available and is user-friendly [85]. Liver ultrasonography can provide a good estimate of the degree, or the extent of hepatic steatosis that is present, based on a series of ultrasonographical characteristics. Shannon and colleagues [86] demonstrated that liver ultrasonography is a useful tool for quantifying steatosis in pediatric patients who have suspected NAFLD; ultrasonography scores strongly correlate with the grade of steatosis when conducting a liver biopsy. Unfortunately, the diagnostic sensitivity of ultrasonography decreases, either when the liver contains <30% of fat, or in individuals with a BMI of 40 or more [87]. Furthermore, ultrasonography cannot rule out the presence of steatohepatitis or fibrosis. Overall the sensitivity of ultrasound in NAFLD ranges from 60% to 94%, with specificity from 84% to 100%.

Computed tomography is more specific, but is not used for the screening of fatty liver in obese children for obvious limitations related to radiation exposure. Magnetic resonance imaging (MRI) and 1H-MRS have the greatest accuracy to determine hepatic fat content, but are rarely used due to the high costs.

The Fibroscan® uses transient elastography to evaluate fibrosis. It correlates well with liver fibrosis on histology in studies in both adults and children. In one study conducted by our group, 67 consecutive children and adolescents with NAFLD were studied [88]. Multilevel likelihood ratios were used to explore the whole spectrum of transient elastography measurements in relation to the degree of fibrosis. We demonstrated that values over 5 kPa could predict the presence of fibrosis. More recently, Fitzpatrick and colleagues [89] have shown that Transient Elastography is a reliable tool in distinguishing different stages of liver fibrosis in paediatric patients, even if it is best performed in children with an autoimmune liver disease, and in those post-transplant, than in those with NAFLD with whom fibrosis had to be excluded. Therefore, Fibroscan® can be useful, but it is not yet sufficient enough in non-invasively monitoring the liver disease progression in children.

To summarize, as shown in Table 1, the differential diagnosis of NAFLD should be based first on clinical features, then on noninvasive tests, and finally on liver biopsy. Other causes of chronic liver disease including hepatitis B and C, Wilson disease, alpha-1-antitrypsin deficiency, autoimmune hepatitis, cystic fibrosis and drug toxicity, and should always be excluded. Alcohol abuse must always be questioned. Especially in young children (less than 3 years), a detailed diagnosis for metabolic causes of fatty infiltrations in the liver should be performed (Table 1).

### Prevention and treatment

Because of the limited knowledge of the molecular pathogenesis of NAFLD, the current therapeutic modalities consist of strategies aimed at decreasing the incidence of the known risk factors. Interventions including community involvement are useful in improving health at individual and public health level. Prevention and control of modifiable risk factors as overweight and unhealthy lifestyle, along with primordial prevention by good pregnancy care -for prevention of low birth weight- and encouraging breast feeding, can impact the overall health of children and adolescents as well as the prevention and control of pediatric NAFLD and the related metabolic syndrome.

**Figure 2:**
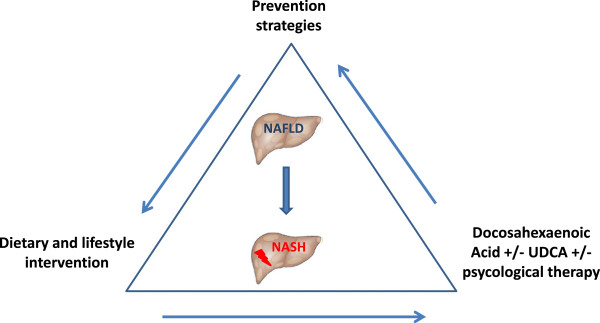
**Proposed treatment schedule in pediatric NAFLD.** Multi-targeted therapy could be the way forward for treating children with NAFLD. Such therapy consists of combinations of the therapeutic approaches directed against various potential targets added to intervention on lifestyle and diet. Psychological therapies could improve adherence to therapies.

The few existing therapeutic strategies are typically not based on drugs, and currently there is no treatment targeting the disease per se. Lifestyle changes and pharmacological treatment have been deeply discussed elsewhere [90]. However, several international groups, based on new pathogenetic paradigms, have recently launched novel clinical trials, combining non-pharmacologic and pharmacological approaches, in an innovative multi-targeted plan. Therapies that simultaneously target the clinical features of NAFLD and NASH (such as dietary interventions to reduce obesity) and molecular mechanisms (such as agents that are effective against the secondary injuries or insults as described above) could be employed. We seriously consider that multi-targeted therapy could be the way forward for treating these children (Figure 2). Such therapy consists of combinations of the therapeutic approaches directed against various potential targets. Recently, interesting dietary supplements such as probiotics and long-chain omega-3 polyunsaturated fatty acids have been adopted in adults with NAFLD [91,92]. Interestingly, these dietary supplements, although considered pharmacological interventions, are often based on natural compounds present in specific foods (yogurt, fish oil, etc.). Among the pathogenetic factors leading to NAFLD, the persistent crosstalk among the gut, the immune system, and the liver, plays a pivotal role [93]. In fact, it is now accepted that specific nutrients increase the intestinal permeability to bacterial endotoxins, activating an immune-mediated inflammatory response of liver resident cells, leading to a profibrogenic phenotype [94]. One recent study on animal models [95], has demonstrated a pivotal role of restoring gut microflora in protecting the liver from fat and preventing cardiovascular disease. As demonstrated in genetic animal models with dyslipidemia, low grade intestinal inflammation can drive steatosis to steatohepatitis. Performing an intervention on intestinal microbiota using probiotics can modulate the expression of nuclear receptors, correcting insulin resistance in the liver and the adipose tissues and protecting against the development of steatohepatitis. Randomized placebo controlled trials on the use of probiotics in NAFLD are ongoing in humans.

Furthermore, studies in experimental models have shown that long-chain omega-3 fatty acids, known as important regulators of hepatic gene transcription, can decrease liver steatosis, improve insulin sensitivity, and decrease markers of inflammation [92]. Recently, Nobili et al. [96] reported the results of a randomized clinical trial based on the use of an omega-3 fatty acid, docosahexaenoic acid. A 6-months docosahexaenoic acid supplementation to the diet, together with physical exercise, ameliorated the body mass index, the insulin sensitivity index, serum ALT and triacylglycerol levels, and steatosis at ultrasound.

## Review

In several recently published works, in large cohorts of overweight and obese children and adolescents from both the western and eastern worlds, NAFLD has emerged to be extremely prevalent and predicted by obesity and male gender. This data underlines the epidemiological dimension of this obesity-related morbidity, even in childhood, inducing one to recommend at least ALT as a screening parameter in basic care. Once NAFLD is diagnosed, it is nowadays compulsory to prescribe lifestyle changes and dietary interventions to reduce pediatric obesity, but also to consider new treatment approaches that simultaneously target the clinical features and *i)* the molecular mechanisms (such as agents that are effective against the secondary injuries or insults) *ii)* the crosstalk among the gut, the immune system, and the liver, in view of the so called NAFLD multi-targeted therapy.

## Conclusions

An early recognition of NAFLD may help when intervening at a younger age, which may reduce future morbidity and mortality from the disease. As a matter of fact, the effects of a modern low-cost diet (with high-fat, high-sugar, high-salt, energy-dense and micronutrient-poor foods) will make children experience, in the next 10–20 years, the highest incidence of an “obesity epidemic” and obesity-related diseases. This has prompted a new sense of urgency, to find novel and early prevention strategies during childhood, to start an efficient primary prevention process.

## Abbreviations

(NAFLD): Nonalcoholic fatty liver disease; (NASH): Non-alcoholic steatohepatatis; (AST): Aspartate amino¬transferase; (ALT): Alanine aminotransferase; (BMI): Body mass index; (MS): Metabolic syndrome; (FFA): Free-fatty-acids; (TG): Triglycerides; (ROS): Reactive oxygen species; (TNF-alpha): Tumor Necrosis Factor alpha; (CVD): Cardiovascular disease; (PNPLA3): Patatin-like phospholipase domain-containing 3; (APOC3): Apolipoprotein C3; (CB2): Cannabinoid Receptor type 2; (SMA): Smooth muscle antibody; (PNFI): Pediatric NAFLD fibrosis index; (ELF): Enhanced liver fibrosis.

## Competing interest

The authors declare that they have no competing interests.

## Authors’ contributions

VG – performed literature searching, wrote the first draft of manuscript with table and figures. FP, FG – performed literature searching. VN – was the guarantor of manuscript, supervised, revised the manuscript. All authors read and approved the final manuscript.

## Pre-publication history

The pre-publication history for this paper can be accessed here:

http://www.biomedcentral.com/1471-2431/13/40/prepub
